# Thyroid Autoimmunity in Vitiligo: A Case-Control Study

**DOI:** 10.7759/cureus.34031

**Published:** 2023-01-21

**Authors:** Praveen Prashant, Renu Garg, Piyush Bansal, Sonia Praveen

**Affiliations:** 1 Department of Biochemistry, Pandit Bhagwat Dayal Sharma Post Graduate Institute of Medical Sciences, Rohtak, IND; 2 Department of Biochemistry, Bhagat Phool Singh Government Medical College for Women, Sonepat, IND; 3 Department of Dermatology, Dr. Sonia’s Skin Clinic, Rewari, IND

**Keywords:** anti-tg, anti-tpo, anti-thyroid peroxidase, anti-thyroglobulin antibody, case-control studies, thyroid antibodies, vitiligo, autoimmunity, thyroid

## Abstract

Introduction

There is scanty evidence regarding the role of autoimmunity in vitiligo, especially in the Asian population. Moreover, the existing studies reported conflicting results. This prompted the investigators to identify the association of thyroid autoimmunity with vitiligo by employing a case-control design in this setting.

Methodology

The present study was a hospital-based case-control study conducted in one of the tertiary care hospitals of North India. We recruited 30 subjects aged 16-60 years with vitiligo attending the skin and venereal diseases outpatient department. The subjects attending the general medicine outpatient department without having a diagnosis of vitiligo were considered for the control group. Thyroid hormones (FT3 and FT4), thyroid-stimulating hormones, anti-thyroid peroxidase (anti-TPO) antibodies, and anti-thyroglobulin (anti-TG) antibodies were the primary investigations performed among the study subjects.

Results

The mean age of the study subjects was 31.3 (SD: 13.3) years. Both the case and control groups were comparable based on selected socio-demographic variables (p > 0.05). There was a statistically significant difference in terms of mean anti-TPO and anti-TG values between the case and control groups in which subjects with vitiligo reported significantly higher values (p < 0.05).

Conclusion

Our study reported a significant elevation in the mean values of the thyroid antibodies (anti-TG and anti-TPO antibodies) in vitiligo subjects compared to control subjects in this setting. Hence, screening for autoimmune thyroid diseases among patients with vitiligo is suggested for the early detection and the initiation of appropriate intervention.

## Introduction

The incidence of skin disorders is increasing exponentially in the population. The skin outpatient department encounters dermatological diseases with either a direct or associated autoimmune etiology. Skin manifestations can be a result of the underlying pathology in the body. Autoimmune disorders of the skin can be clinically diagnosed and confirmed using various biochemical markers [1]. In the case of organ-specific autoimmune diseases, a single organ or gland is targeted in response to a unique antigen. The manifestations of the disease are primarily limited to that organ. Humoral or cell-mediated effector mechanisms are responsible for target organ damage. The antibodies may stimulate or block normal tissue function. Direct cellular damage is also relevant for some autoimmune diseases. When lymphocytes or antibodies bind to cell-membrane antigens, it results in cell lysis and an inflammatory response, after which the damaged cellular structure is gradually replaced by the healing connective tissue. It may result in tissue scarring and a decline in organ function [2].

Anti-thyroglobulin (anti-TG) antibody (Ab) was the first antibody reported to be associated with autoimmune thyroid disease (AITD). Most anti-TG Abs are of the immunoglobulin class IgG, which is more prevalent than IgA. It is an intra-follicular antibody that binds to immune cells and antigens, potentially resulting in tissue destruction in certain cases. Massive destruction of thyroid gland tissue induces structural changes in thyroglobulin (TG), resulting in antibody production against TG [3-5]. The highly sensitive and specific assays for serum autoantibodies and recombinant technology led to the use of thyroid peroxidase (TPO) in molecular cloning. The importance of the role of thyroid lymphocytes is evident from the high frequency of TPO autoantibody characterization. These autoantibodies for autoimmune thyroiditis are also found in the serum at microgram- to milligram-per-milliliter concentrations [6-8].

Vitiligo, an acquired skin disease of progressive melanocyte loss, is clinically characterized by well-defined milky-white macules that may also include white hairs or poliosis. Vitiligo is frequently associated with disorders of autoimmune origin, with thyroid abnormalities being the most common [9]. Anti-thyroid peroxidase (anti-TPO) Ab was significantly more common in vitiligo patients, especially in young women, as this antibody is a relatively sensitive and specific marker of autoimmune thyroid disorders, including Hashimoto's thyroiditis (HT) and Graves' disease [10].

There is scant evidence regarding the role of autoimmunity in vitiligo, especially in the Asian population. Moreover, the existing studies reported conflicting results. It prompted the investigators to identify the association of thyroid autoimmunity with vitiligo by employing a case-control design in this setting.

## Materials and methods

The present research was a hospital-based case-control study conducted in the Department of Biochemistry in collaboration with the Department of Skin and Venereal Diseases at Bhagat Phool Singh Government Medical College for Women, Khanpur Kalan, Sonepat, Haryana, India. The sample size of 60 was decided for the study, with 30 subjects each in the case and control arms, and it was calculated using the online "Epi Info" sample size calculator tool (CDC, Atlanta, GA). The duration of the study was determined to be one year. Pregnant females, subjects with known thyroid disease, thyroid surgery, thyroid medication, acute or chronic systemic illness, and subjects with other autoimmune diseases that may mimic or hamper the results were excluded from the study. The Institutional Scientific Research and Ethics Committee approved the study plan after the due procedure.

Data collection process

After obtaining informed, voluntary written consent, subjects were recruited as per the inclusion and exclusion criteria into the case or control groups. The purpose of the study was explained in vernacular to the study participants, and a patient information sheet was also provided to them. After obtaining voluntary consent, the subjects with clinically diagnosed vitiligo were included in the case group (group A), and age-matched healthy subjects were included in the control group (group B).

Subjects were instructed to remain fasting overnight for eight to 10 hours before the blood sample collection. A 5 ml blood sample was collected aseptically from the antecubital vein following the standard protocol by the phlebotomist in red-colored vacuum tubes. The blood sample was processed and analyzed on the same day. The biochemical parameters of interest were estimated using commercially available enzyme-linked immunosorbent assay (ELISA) kits. Internal quality control was also maintained, along with an External Quality Assurance Scheme sourced from Christian Medical College, Vellore, Tamil Nadu, India, for quality control.

Statistical analysis

We compared the characteristics of various data using Pearson's chi-square test and an independent sample t-test using SPSS version 26.0 (IBM Corp., Armonk, NY). The significance level (p-value) was taken as <0.05.

## Results

Socio-demographic profile

The mean age of the vitiligo subjects was 31.3 (SD = 13.3) years as compared to 32.1 (SD = 11.6) years in the control group. In the vitiligo group, 60% of subjects were aged less than 25 years compared to 53.3% in the control group, reflecting similar age distribution. The p-value was calculated using the chi-square test, and the difference was not significant (P = 0.79). The case and control groups had 13 and 19 males, respectively, and on the comparison, it was also found not significant on the chi-square test (P = 0.11). Most of the subjects in both groups had a rural background, and the distribution was not significant in the comparison (P = 0.43; Table 1).

**Table 1:** Comparison of socio-demographic variables in the case and control groups

Variable	Cases (N)	Control (N)	Chi-square value	P-value
Age in years (mean age/SD)	31.3/13.3	32.1/11.6	0.6	0.79
Less than 25	18	16		
More than 25	12	14		
Gender				
Male	13	19	2.4	0.11
Female	17	11		
Place of residence				
Rural	19	16	0.6	0.43
Urban	11	14		

Comparison of biochemical parameters in the case and control groups

A comparison of the thyroid hormone profile revealed a significant increase in the mean values of serum FT3 and serum FT4 in the subjects in the control group compared to the vitiligo cases (p < 0.05). The mean values of serum FT3 were 2.8 (SD = 0.7) and 3.3 (SD = 0.4) ng/mL in the case and control groups, respectively. The mean value of serum FT4 in group A was 1.0 (SD = 0.4) pg/mL, and in group B, it was 1.2 (SD = 0.3) pg/mL. Although the mean serum thyroid-stimulating hormone (TSH) value (in mU/mL) was higher among subjects in the control group (2.62, SD = 1.5) than in the cases (3.02, SD = 0.93), the association did not find statistical significance (p > 0.05). There was a wide range of standard deviation in both anti-TPO and anti-TG values in the study groups, and the data were not normally distributed, so non-parametric tests were recruited to compare the data. The mean serum anti-TPO Ab levels (in IU/mL) were 210.5 (SD = 259.62) in group A, while they were 11.39 (SD = 28.42) in group B. The mean serum anti-TG Ab levels (in IU/mL) were 72.46 (SD = 109.33) in the case group and 2.49 (SD = 9.05) in the control group, respectively. There were statistically significant differences in mean serum anti-TPO and serum anti-TG Abs levels between the case and control groups on the Mann-Whitney test (p < 0.05; Table 2). Based on serum TSH levels, the subjects were categorized as euthyroid, hypothyroid, and subclinical hypothyroid. Out of 26 euthyroid subjects in the vitiligo group, 10 tested positive for one of the antibodies, while four had subclinical hypothyroidism. Only one subject was in subclinical hypothyroidism in the control group, while the rest were euthyroid.

**Table 2:** Comparison of the study parameters in the case and control groups * P-value < 0.05 is significant, Mann-Whitney test. TSH: thyroid-stimulating hormone; anti-TPO: anti-thyroid peroxidase; anti-TG: anti-thyroglobulin; Ab: antibody.

Variables with reference range	Cases (mean/SD)	Control (mean/SD)	t-value	P-value
Serum FT3 (2.4-4.2) (pg/mL)	2.8/0.7	3.3/0.4	2.9	0.005*
Serum FT4 (0.7-1.3) (ng/mL)	1/0.4	1.2/0.3	2.3	0.02*
Serum TSH (0.3-4.3) (mU/L)	2.6/1.5	3/0.6	1.5	0.13
Serum anti-TPO Ab (>50) (IU/mL)	210.5/259.6	10.9/29.3	4.1	0.01*
Serum anti-TG Ab (>100) (IU/mL)	72.4/109.3	0.98/3.1	3.5	0.01*

## Discussion

AITDs are commonly encountered in clinics worldwide. They can be clinically diagnosed with confirmatory biochemical or histological studies and radiological findings. Given the multi-modal domain of autoimmune diseases, the index study explored the association of dermatological disorders with AITDs. The present study was a hospital-based case-control study conducted in one of the tertiary care hospitals in North India. Thirty vitiligo patients aged 16-60 years visited the skin and venereal diseases outpatient department. The subjects who visited the general medicine outpatient department without a diagnosis of vitiligo were considered for the control group. Thyroid hormones (serum T3 and serum T4), serum TSH, anti-TPO Ab, and anti-TG Ab were the primary investigations performed among the study subjects.

Our study reported an increased prevalence of AITDs in vitiligo subjects compared to control subjects. The present study observed a significant elevation in the mean values of the thyroid antibodies (anti-TG and anti-TPO Abs), suggesting a strong association between AITDs and vitiligo. Schunter et al. genotyped 281 patients with variable autoimmune endocrinopathies, including HT, Graves' disease, type 1 diabetes, Addison's disease, autoimmune polyglandular syndrome (APS), and vitiligo, along with 1858 controls, and found a correlation with forkhead transcription factor D3 (FoxD3), a gene involved in embryonal melanogenesis. Patients with vitiligo were found to have a higher frequency of the risk allele FoxD3 (30%) than healthy controls (18.2%). It was also seen that the variant was associated with the incidence of elevated anti-TPO and anti-TG antibodies [11]. Another study by Alawneh et al. reported an 88.5% prevalence of AITDs in a total of 130 vitiligo patients in Jordan. In contrast, positivity was 9.1% for anti-TG Ab and 11.1% for anti-TPO Ab in the 99 control subjects [12]. A study by Philip et al. reported 92% positivity for anti-TPO Ab and 66% for anti-TG Ab in 63 HT subjects. The cutaneous manifestations of HT were investigated in the study, and it was found that three subjects included in the study were also suffering from vitiligo. Each individual was anti-TPO and anti-TG positive [13]. A study conducted among 98 vitiligo subjects estimated the prevalence of anti-TPO Ab in 34.7% of disease subjects [14]. The results of these studies were consistent with our findings.

Our study showed a significant rise in mean anti-TPO and anti-TG antibody levels among vitiligo patients. Similar results were also reported in earlier studies. Gopal et al. recruited 150 subjects with vitiligo and 100 control subjects and reported anti-TPO and anti-TG Ab positivity in 17 cases in contrast to eight control subjects (11.34% and 5.34%, respectively). They concluded that a clear association between vitiligo, autoimmune hypothyroidism, and diabetes mellitus persists; further, they proposed that vitiligo shares a common genetic link with these autoimmune disorders [15]. These results were aligned with those of our study, though we did not compare vitiligo with other comorbidities. In a study by Colucci et al., 79 subjects with vitiligo and 100 controls were recruited and evaluated for autoimmune thyroid status. The researchers identified that 77 out of the 79 subjects were positive for the antibodies, while only one control subject was positive [16]. In line with our study findings, Moradi et al. recruited 100 vitiligo subjects and found anti-TPO and anti-TG Ab positivity rates of 36.7% and 32.1%, respectively. Hypothyroidism was also found in 16.1% of subjects, concluding a role for thyroid autoimmunity in vitiligo and associated hypothyroidism [17]. These studies suggest the role of thyroid dysfunction in the etiopathogenesis of vitiligo. A few studies reported the prevalence of autoimmunity to be lower than our study, while a few others found an association between thyroid autoimmunity and vitiligo, which was, however, not statistically significant. Kroon et al. recruited 260 children and adolescents and calculated anti-TPO Ab positivity in 27 out of the 260 subjects, i.e., 10.5% [18].

Another study by Kurtipek et al. in Turkey reported a 14.8% prevalence of AITDs in 108 study patients with vitiligo (anti-TPO Ab in 16 and anti-TG Ab in nine patients). They concluded that the frequency of autoantibodies was low in vitiligo subjects [19]. The exact mechanisms thought to play a role in the etiopathogenesis of vitiligo are described at length in the literature. The main proposed and accepted hypotheses are genetic, humoral immunity, cellular immunity, neural hypothesis, the free radical injury theory, melanocyte injury, heavy metal toxicity, and autoimmunity [20]. The present study elucidates the association of thyroid autoimmunity among patients with vitiligo in the Indian setting.

Limitations

The small sample size resulted in skewed mean values due to extreme outliers. A larger sample size could have overcome this limitation. A longitudinal cohort study could have provided a higher level of evidence; however, it was not undertaken due to the coronavirus disease 2019 (COVID-19) outbreak in this setting.

## Conclusions

High anti-TPO and anti-TG Abs levels are associated with AITD. The significant rise in levels of these antibodies in vitiligo patients is suggestive of the association of AITD with vitiligo. These antibodies can be detected even in euthyroid or subclinical hypothyroid patients, leading later to the destruction of the thyroid gland. In addition, it is hypothesized that early detection of these antibodies may be helpful in vitiligo patients for initiating appropriate intervention. More studies are needed for a better understanding of the course of the disease.
